# Endogenous Hormone Profile and Sugars Display Differential Distribution in Leaves and Pseudobulbs of *Laelia anceps* Plants Induced and Non-Induced to Flowering by Exogenous Gibberellic Acid

**DOI:** 10.3390/plants11070845

**Published:** 2022-03-23

**Authors:** Olga Tejeda-Sartorius, Ramón Marcos Soto-Hernández, Rubén San Miguel-Chávez, Libia Iris Trejo-Téllez, Humberto Caamal-Velázquez

**Affiliations:** 1 Campus Montecillo, College of Postgraduates in Agricultural Sciences, Texcoco 56230, Mexico; msoto@colpos.mx (R.M.S.-H.); sanmi@colpos.mx (R.S.M.-C.); tlibia@colpos.mx (L.I.T.-T.); 2 Campus Campeche, College of Postgraduates in Agricultural Sciences, Champotón 24450, Campeche, Mexico; hcaamal@colpos.mx

**Keywords:** back and current growth structure linked, flowering, Orchidaceae, plant growth regulators (PGRs), orchid vegetative organs

## Abstract

A profile of endogenous hormones and sugars in leaves and pseudobulbs of *Laelia anceps* subsp. *anceps* (Orchidaceae) plants induced and non-induced to flowering by the effect of different doses of exogenous gibberellic acid (GA_3_), considering the current and back growth structures (CGS and BGS), were investigated. A factorial experiment with five doses of GA_3_ and two growth structures was designed. Adult plants with undifferentiated vegetative buds were selected and sprayed with doses of 0, 400, 600, 800, and 1000 mg GA_3_ L^−1^. The main results showed a strong interaction between GA_3_ dose and growth structures, which promoted the highest kinetin (KIN) concentration in CGS. Exogenous GA_3_ increased endogenous GA_3_ in leaves and pseudobulbs induced (I-Leaf and I-PSB) and non-induced (NI-Leaf and NI-PSB) to flowering. For sugar concentration, the 400 mg L^−1^ GA_3_ dose promotes significant interaction with the CGS in NI-PSB. In general, the hormone profile revealed opposite balances of endogenous hormone concentrations for KIN, zeatin (ZEA), *trans*-zeatin (T-ZEA), indoleacetic acid (IAA), indole-3-butyric acid (IBA) and GA_3_, not only for growth structures but also for vegetative organs analyzed, depending on whether the plants were induced or not induced to flowering, with the highest concentration of endogenous hormones in pseudobulbs. Likewise, different sugar concentration balances were observed. These balances of both endogenous hormones and sugars are likely to be involved in the flowering of *L. anceps*.

## 1. Introduction

With an estimated 19,000 to 25,000 species [1,2], the Orchidaceae family is one of the largest and most diverse of the angiosperms, and its blooms have captivated and intrigued the world. However, the mechanisms that cause flower induction in orchids are complex, with many unanswered questions [3]. Orchids generally take several years to mature from their vegetative state to their reproductive stage. As with most flowering plants, once they reach reproductive maturity, their flowering is influenced by external factors (that is, photoperiod and temperature) as well as endogenous pathways (that is, genes and hormones), primarily divided into two stages: transition and flower development [4]. Most orchids have defined favorable seasons for floral induction, inflorescence, and flower development based on their natural habitats [4,5].

In plants, several physiological studies have revealed that certain compounds and processes, such as the role of sugars and gibberellins (GAs), play a role in the floral transition [6]. On the one hand, changes in sugar concentration affect growth and development processes, such as cell division, vegetative growth, flowering, and aging. Sugar-induced signal transduction pathways interact with other pathways in plant tissues, such as hormonal pathways, to form a complex communication and signaling network that controls the aforementioned development phases [7,8]. Sucrose is an essential component of the “flower stimulus” in most species, according to evidence [9], and it can function in long-distance signaling during flower induction [6]. On the other hand, endogenous, also known as “autonomous”, pathways and GAs act independently of environmental influences [10,11]. One of the flowering pathways in plants is the gibberellic acid (GA) signaling pathway [12,13].

Although the role of GAs in the process of transition to flowering is difficult to establish, several studies show the role of endogenous GAs in the flowering process [6]. Furthermore, when plant growth regulators (PGRs) are applied exogenously, they can affect the hormonal balance of the treated plants, either through natural hormones or their synthetic analogues, by inhibiting the biosynthesis of endogenous hormones or their translocation from the site of production to the site of action, as well as by blocking hormone receptors [14]. In this way, some of the main exogenously applied PGRs involved in plant flowering are gibberellins (GAs) [13,14]. Among the GAs identified, GA_1_, GA_3_, GA_4_, and GA_7_ are considered the most common biologically active forms [13], but GA_3_ is the most widely used gibberellin, among others, to increase flowering in certain species [15].

There is important information about the application of GAs and their effects on endogenous composition during flower induction [6,13,16]. In this context, Zhang et al. [13] showed that GA_3_ spraying significantly increased the content of endogenous GAs and that of zeatin-riboside (ZR) was reduced at 44 days after full bloom, which could negatively affect apple flowering (*Malus domestica*). In addition, Guan et al. [17] reported that exogenous GA_3_ application significantly promoted flower bud development and also stimulated the synthesis of endogenous GA_3_ and IAA but reduced ABA levels in tree peony (*Paeonia suffruticosa*). Nevertheless, limited literature reports the effect of exogenous GA_3_ in endogenous hormone levels for orchids, although Su et al. [18] investigated GA_3_ treatments in *Phalaenopsis* plants, suggesting that aspects of flower bud initiation and flowering development are closely associated with increases in endogenous GAs, even in GA_3_-treated plants.

Epiphytic orchids are thought to be an interesting model for plant development and metabolic studies because they may exhibit unusual hormonal control during stem and root growth [19]. However, little is known about the hormonal distribution in leaves and pseudobulbs and its association with flowering process. Orchids in the reproductive stage may or may not initiate the next flowering cycle, and the mechanisms underlying this phenomenon are not fully understood, particularly in species with sympodial growth in which each annual growth structure, composed of a leaf and a pseudobulb, is potentially inductive to flowering.

Based on previous information, this work aims to analyze the effect of different doses of gibberellic acid (GA_3_) on the endogenous hormone profile and sugars in leaves and pseudobulbs of *Laelia anceps* subsp. *anceps* plants induced and non-induced to flowering as well as their distribution in current and back growth structures. This research attempts to provide information that leads to a better understanding on flowering of this species.

## 2. Results

### 2.1. Endogenous Hormonal Concentration by Treatment Effect

**Figure 1:**
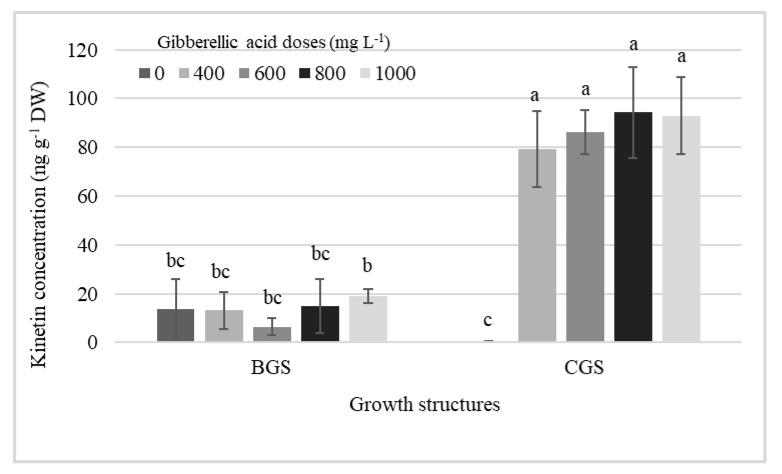
Interaction effects among different doses of exogenous gibberellic acid (GA_3_; mg L^−1^) and the back and current growth structure (BGS, CGS) on the concentration of KIN in pseudobulbs induced to flowering of *Laelia anceps* subsp. *anceps* plants. Bars with different letters + SD indicate significant statistical differences (Tukey, *p* ≤ 0.05, *n* = 3). DW = dry weight.

Table 1 shows the significant effect of factors on endogenous hormone concentration in leaf and pseudobulb for plants that were induced and not to flowering. The analysis revealed differences in cytokinins (CKs), auxins, and GA, primarily in growth structure (GS), but, in addition, KIN and endogenous GA_3_ showed differences by exogenous GA_3_ dose in the pseudobulb induced to flowering (I-PSB). Significant effects of single factors, GA_3_ doses and GS, and their interaction on KIN concentration in I-PSB were observed (Table 1, Figure 1).

GA_3_ exogenous doses and its interaction with the growth structures increased the endogenous KIN concentration in I-PSB CGS, which was higher (99%) than when GA_3_ was not used, and 86% higher than the average of all KIN concentrations in BGS (Figure 1).

**Figure 2:**
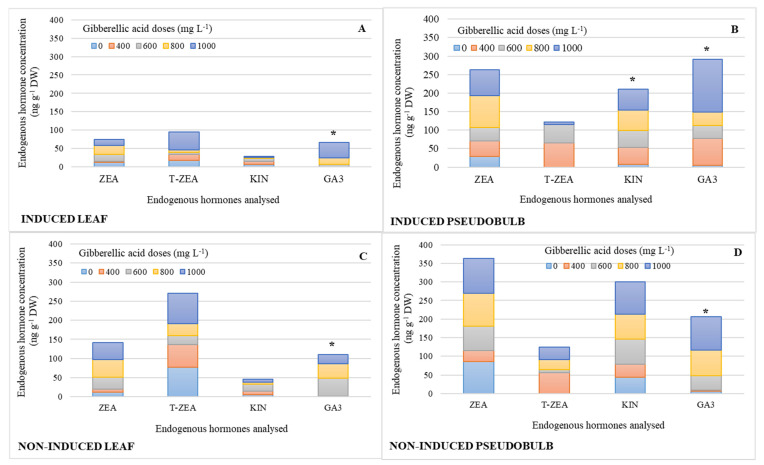
Endogenous hormone concentration of zeatin (ZEA), trans-zeatin (T-ZEA), kinetin (KIN), and gibberellic acid (GA_3_), in leaf and pseudobulb of *Laelia anceps* subsp. *anceps* plants, induced (**A**,**B**) and non-induced (**C**,**D**) to flowering by different doses of exogenous gibberellic acid (GA_3_; mg L^−1^) (The bars with asterisks show the endogenous hormone with significant differences, which are shown in Table 2; *n* = 3).

Figure 2 shows the endogenous hormone profile in leaf and pseudobulb by simple effect of GA_3_ exogenous doses. KIN and GA concentrations significantly increased in I-PSB by 87 and 96%, respectively, compared to the treatment without GA_3_ (Figure 2B), while in the pseudobulb non-induced to flowering (NI-PSB), a 1000 mg L^−1^ GA_3_ dose promoted 95% higher concentration than the lowest doses (Figure 2D). In the induced leaf (I-Leaf), 1000 mgL^−1^ GA_3_ was significantly higher than the three lowest doses by 94%; and in the non-induced leaf (NI-Leaf), the 600 mg L^−1^ GA_3_ dose was also significantly higher than the two lowest doses by 98%. Table 2 displays the means of the endogenous hormones with statistically significant differences indicated in the Figure 2.

It is worth noting that the ratio of endogenous ZEA, KIN, and GA_3_ concentrations in pseudobulb was 68% higher (Figure 2B,D) than in leaf (Figure 2A,C), regardless of whether plants were induced to flowering or not, except for T-ZEA, which is 54% higher in NI-Leaf relative to pseudobulb concentration.

ZEA and T-ZEA in NI-Leaf double their counterparts in I-Leaf (Figure 2C).

Similarly, ZEA and KIN concentrations were 20 and 38% higher in NI-PSB, respectively (Figure 2D) than in I-PSB, while GA_3_ was 33% higher in I-PSB (Figure 2B).

**Figure 3:**
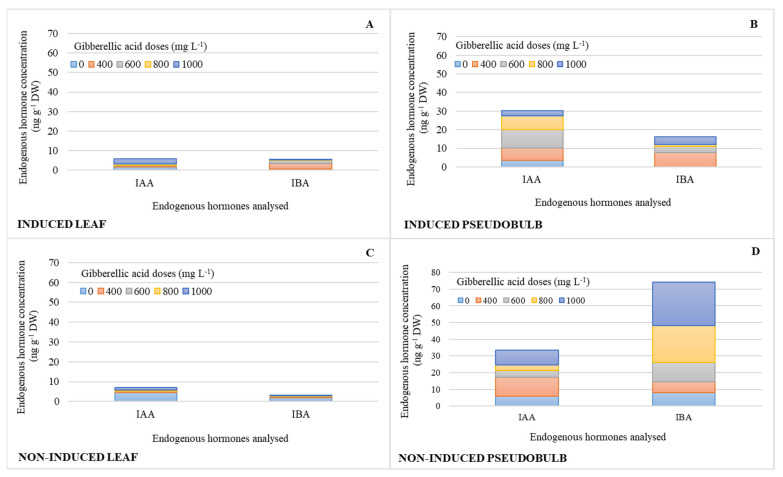
Endogenous hormone concentration (*n* = 3) of indoleacetic acid (IAA) and indole-3-butyric acid (IBA) in leaf and pseudobulb of *Laelia anceps* subsp. *anceps* plants, induced (**A**,**B**) and non-induced (**C**,**D**) to flowering by different doses of exogenous gibberellic acid (GA_3_) (There were no statistical differences in these data, thus only the means of the concentrations are presented).

The concentrations of auxins (IAA, IBA) were similar in I-Leaf and NI-Leaf. Regardless of whether they were induced to flower, IAA and IBA increased their pseudobulb concentration by 73 and 90%, respectively (Figure 3B,D), compared to the leaf concentration (Figure 3A,C). It is also observed that the action of the 800 and 1000 mg L^−1^ doses increases the IBA concentrations in NI-PSB by 67 and 83%, respectively, compared to the other doses, and 72% more (Figure 3D) than I-PSB (Figure 3B), while ABA levels were in the range of 0.08 for leaf and 0.2 ng g^−1^ DW for pseudobulb.

### 2.2. Endogenous Hormonal Concentration by Distribution in Growth Structure

**Figure 4:**
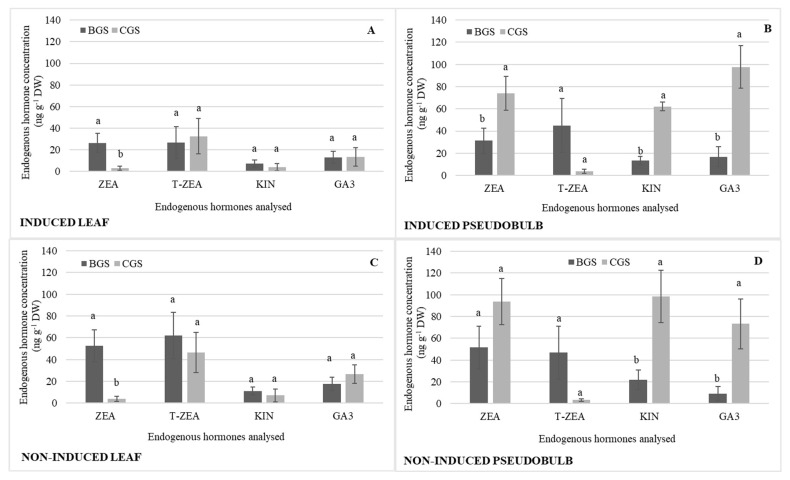
Endogenous hormone concentration of zeatin (ZEA), trans-zeatin (T-ZEA), kinetin (KIN), and endogenous gibberellic acid (GA_3_) in the back and current growth structure (BGS, CGS) of leaf and pseudobulb induced (**A**,**B**) and non-induced (**C**,**D**) to flowering in *Laelia anceps* subsp. *anceps* plants, by different doses of exogenous gibberellic acid (GA_3_). Bars with different letters + SD indicate significant statistical differences (Tukey, *p* ≤ 0.05; *n* = 3). DW = dry weight.

There were consistent statistical differences in back and current growth structures (BGS and CGS) related to different endogenous hormones, as shown in Table 1. ZEA showed statistical differences in I-Leaf and NI-Leaf, and its concentration was 56% higher in BGS compared to CGS (Figure 4A,C). T-ZEA was 61% higher in NI-Leaf BGS (Figure 4C) in comparison to I-Leaf BGS (Figure 4A). ZEA, KIN, and GA showed statistical significance between CGS and BGS of I-PSB and NI-PSB, with CGS being higher regardless of flowering induction or not (Figure 4B,D). KIN and GA, nevertheless, reverse their concentration depending on their reproductive stage. Thus, GA is 30% higher in I-PSB CGS than in NI-PSB CGS, while KIN is 34% higher in NI-PSB than in I-PSB (Figure 4B,D).

**Figure 5:**
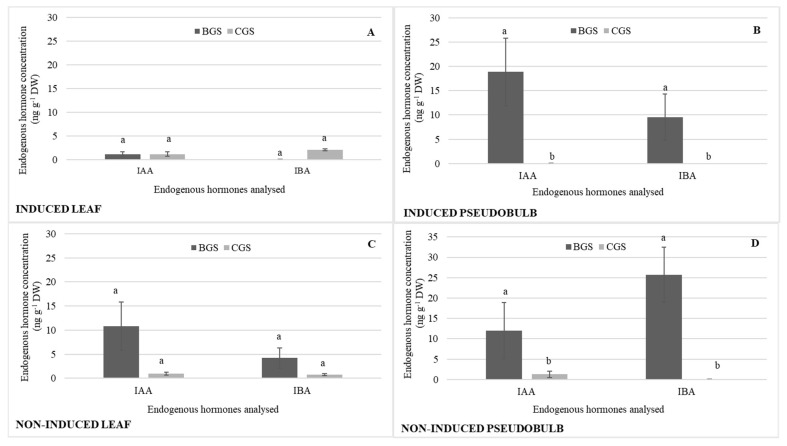
Endogenous hormone concentration of indole acetic acid (IAA) and indole-3-butyric acid (IBA) in the back and current growth structure (BGS, CGS) of leaf and pseudobulb, induced (**A**,**B**) and non-induced (**C**,**D**) to flowering in *Laelia anceps* subsp. *anceps* plants, by different doses of exogenous gibberellic acid (GA_3_). Bars with different letters + SD indicate significant statistical differences (Tukey, *p* ≤ 0.05; *n* = 3).

In terms of auxins, it highlights that the IAA concentration was 88% higher in NI-Leaf BGS (Figure 5C) compared to I-Leaf BGS (Figure 5A), while IAA and IBA were significantly higher in I-PSB and NI-PSB BGS compared to CGS, and IAA concentration was 27% higher in I-PSB compared to NI-PSB IAA; IBA was 71% higher in NI-PSB BGS compared to I-PSB BGS (Figure 5B,D).

### 2.3. Concentration of Total Sugars

**Figure 6:**
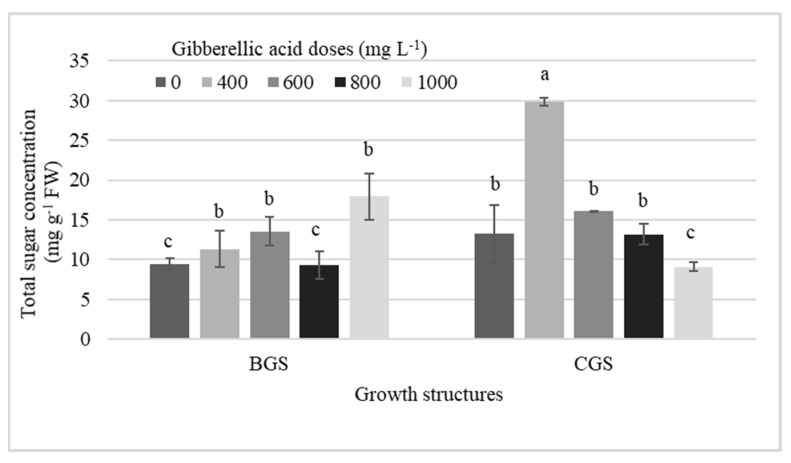
Interaction effects among different doses of exogenous gibberellic acid (GA_3_), and the back and current growth structure (BGS, CGS) on the concentration of total sugars in pseudobulbs of *Laelia anceps* subsp. *anceps* plants non-induced to flowering. Bars with different letters + SD indicate significant statistical differences (Tukey, *p* ≤ 0.05; *n* = 3).

Stronger statistical effects for total sugar concentration were observed in NI-PSB, both for single effects and for their interaction, while single effects by GS and by GA_3_ dose were present in I-Leaf and NI-Leaf, respectively (Table 3). Thus, 400 mg L^−1^ GA_3_ dose outperformed all treatments by an average of 56% (Figure 6).

### 2.4. Total Sugars by Exogenous GA_3_ Dose Effect

**Figure 7:**
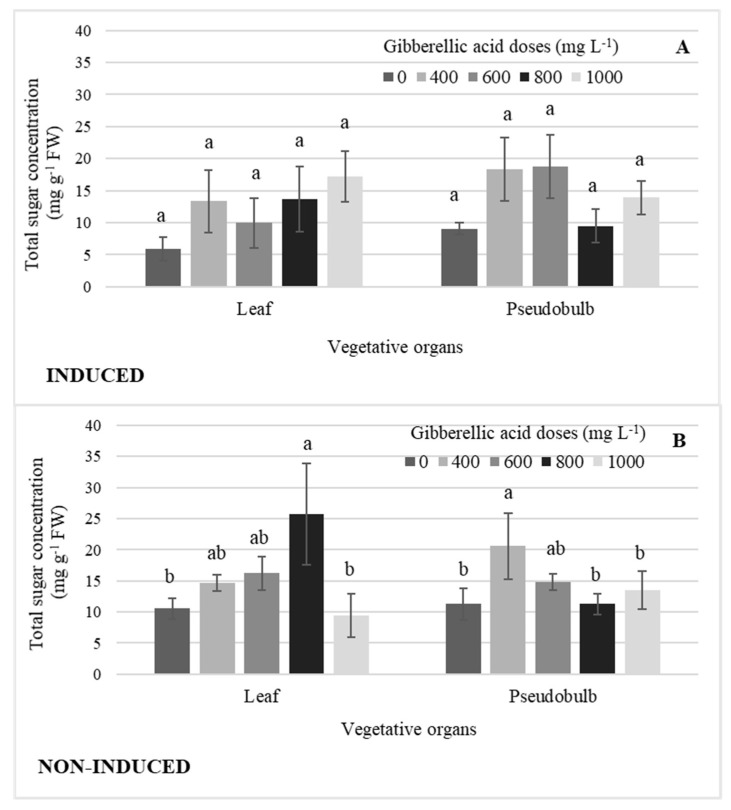
Concentration of total sugars in leaf and pseudobulb, (**A**) in induced and (**B**) non-induced plants of *Laelia anceps* subsp. *anceps* by different doses of exogenous gibberellic acid (GA_3_). Bars with different letters + SD indicate significant statistical differences (Tukey, *p* ≤ 0.05; *n* = 3). FW = fresh weight.

There were no significant differences by GA_3_ dose in leaf and pseudobulb induced flowering (Figure 7A). In NI-Leaf, the 800 mg L^−1^ GA_3_ dose significantly increased the concentration of total sugars by an average of 63% when compared to the highest GA_3_ dose and that without GA_3_, while the 400 mg L^−1^ GA_3_ dose in NI-PSB increased it by 45% (Figure 7B).

### 2.5. Concentration of Total Sugars by Distribution in Growth Structures

**Figure 8:**
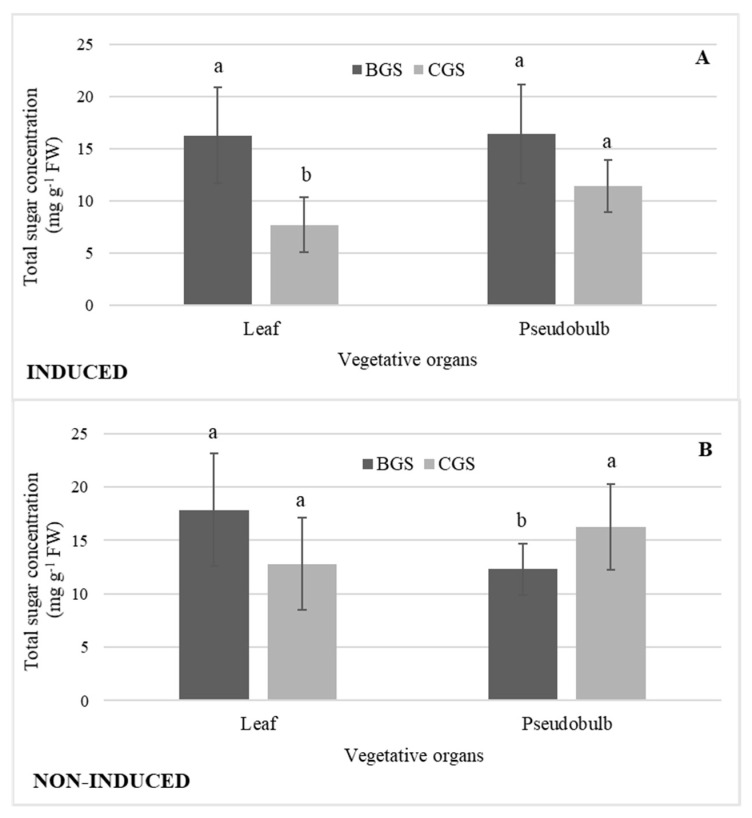
Concentration of total sugars in leaf and pseudobulb of back and current growth structure (BGS, CGS) of *Laelia anceps* subsp. *anceps* plants, (**A**) induced and (**B**) non-induced to flowering. Bars with different letters + SD indicate significant statistical differences (Tukey, *p* ≤ 0.05; *n* = 3).

There was the highest concentration of total sugars in I-Leaf and NI-Leaf BGS; however, in NI-Leaf there was a higher concentration in both BGS (20%) and CGS (36%) in comparison to the concentrations in I-Leaf. The sugars concentration of I-PSB was 19% higher in BGS than in CGS, but in NI-PSB there was a reverse distribution, with the concentration statistically higher in CGS (19%). Additionally, NI-PSB CGS had a higher concentration of sugars (20%) than I-PSB of CGS (Figure 8A,B).

## 3. Discussion

### 3.1. Endogenous Hormone

In general, doses between 600 and 1000 mg L^−1^ of exogenous GA_3_ increased the concentration of endogenous GA_3_ (Figure 2, Table 1). Similarly, Su et al. [18] reported that GA_3_ applications in *Phalaenopsis* increased the concentration of endogenous GA_3_. Similar results have also been reported by other authors for different species, such as in apple (*M. domestica*) [13], and in tree peony (*P. suffruticosa*) [17]. In all cases, these results are related to the role of GA_3_ in flowering. In support of this idea, it is important to mention that applications of exogenous GA_3_ have improved some flowering parameters of *L. anceps*, such as reduction of time to anthesis and flower life [20].

Likewise, in the analysis of study factors, a strong effect of GA_3_ dose, growth structure, and their interaction on endogenous KIN concentration was observed for I-PSB, where GA_3_ doses had a superior effect on CGS compared to non-GA_3_ treatment and BGS concentrations (Table 1, Figure 1). Since its isolation several decades ago, KIN (N6-furfuryladenine) has been used as a synthetic cytokinin in several physiological studies in plants and is attributed with strong antioxidant activity and important agricultural and health properties [21,22]. However, the biological significance of endogenous KIN and the molecular mechanisms of its action are not fully understood [22]. Furthermore, its role as an endogenous compound related to flowering was not documented, although it is widely used as an exogenous growth regulator in ornamental plants, with some beneficial effects on flower development, as in *Bougainvillea glabra* var. “Elizabeth Angus” [23], or in *Phalaenopsis* [24]; and in the improvement of vase life, as in *Gladiolus grandiflora* [25], or in the orchid *Oncidium* spp. [26]. The fact that KIN was found in some abundance in the pseudobulbs of *Laelia anceps* and, above all, its evident influence by the action of GA_3_ doses in interaction with the CGS of I-PSB (Figure 1) raises the possibility of its involvement in flowering, as has been generally attributed to cytokinins.

These results are in line with the simple effects analysis, where 800 and 1000 mg L^−1^ doses of GA_3_ increased the endogenous KIN and GA concentration of I-PSB (Figure 2). Although it has been reported that GAs individually applied do not induce flowering [27], Su et al. [18] suggest that flower bud initiation and inflorescence development of *Phalaenopsis* hybrids are closely associated with increases in endogenous GAs, even in GA_3_-treated plants, which is consistent with our findings. In addition, Wen et al. [28] indicated that there is a CKs–GA signaling network, which supports floral initiation in *Dendrobium*. Other authors, as Phengphachanh et al. [29] investigated the effects of long days and GA_3_ on flowering and endogenous hormone levels of *Rhynchostylis gigantea*. The authors reported a decrease in ABA and an increase in t-ZR (trans-zeatin riboside) in leaf and stem related to the initiation of flower buds and early flowering of the species. A relatively different effect was observed in our research, as an inverse balance in T-ZEA between the BGS and CGS of both I-Leaf and NI-Leaf seems to be involved in flowering by inhibiting or inducing it depending on their concentration (Figure 4).

In floral buds, some authors discovered a higher content of gibberellins from the non-13-hydroxylation pathway (GA_9_, GA_7_, and GA_4_) and CKs (particularly isopentenyl-type species), while vegetative buds contained more GAs (mostly from the early 13-hydroxylation pathway) and less CKs [16]. This information differs slightly from the findings of this study in that the structures that remained vegetative (not induced to flowering) had a higher concentration of CKs but a lower concentration of GAs in the NI-PSB. In contrast, it has been reported that high levels of GA_3_ had inhibitory effects on flower formation during induction and initiation periods in olive (*Olea europaea* L.) [30].

Regardless of doses, a higher concentration of endogenous hormones is noticeable in pseudobulb than in leaf, except for T-ZEA which is higher in leaf, as indicated above. However, on the one hand, the concentration of CKs (ZEA and KIN) in NI-PSB is higher than in I-PSB. Our findings could be explained in part for that which was found in *Dendrobium*, where feedback inhibition of endogenous cytokinin levels resulted in an increase in GA signaling, which was important for subsequent floral development [28]. On the other hand, the lower concentration of CKs (ZEA and KIN) in I-PSB was most likely destined for the floral meristem and subsequent flowering development. Some authors analyzed the putative role of CKs in flower induction in *Arabidopsis* (Columbia). They found that isopentenyl adenine forms of cytokinins increased 16 h after the start of the induction treatment (long day) and that, at 30 h, the shoot apical meristem of induced plants contained more CKs (isopentenyl adenine and zeatin) than non-induced controls, whose increase they linked to early events of floral transition [31].

There was a differential behavior in the distribution of BGS and CGS concentrations, with a higher concentration of ZEA and T-ZEA in the NI-Leaf BGS in comparison to I-Leaf. A higher concentration of these hormones in BGS, as discussed below, is likely to influence flowering in conjugation with auxins, depending on a certain balance. In the case of pseudobulbs, however, the ZEA, KIN, and GA concentrations were higher in the CGS of both I-PSB and NI-PSB. However, GA_3_ is 30% higher in I-PSB, but KIN is 34% higher in NI-PSB (Figure 4), i.e., there is an opposite KIN–GA balance in pseudobulbs induced and non-induced to flowering in CGS. With these data, the involvement of GA_3_ in flowering could not be discarded. Like our findings, Zhang et al. [13] investigated exogenous GA_3_-regulated flowering in apple (*Malus domestica*-Borkh.) trees, and discovered that it increased endogenous GAs while decreasing zeatin-riboside (ZR) content. The authors conclude that GA_3_ sprays disrupt the balance of the two hormones, preventing floral induction. According to [32], plant growth and development are influenced by hormone balance or opposing effects, changes in the effective concentration of one hormone by another, and hormone sequential actions. According to Pallardy [33], GA and zeatin are important in the differentiation of the floral primordium and ovule development of *Agapanthus praecox* ssp. *orientalis*. The use of exogenous PGRs revealed that GA signaling regulates scape elongation and stimulates early flowering, according to the authors.

In the case of endogenous auxins, NI-Leaf BGS and I-PSB BGS had higher IAA concentration. Above all, greater IBA concentration in NI-PSB BGS (Figure 5) is outstanding. IBA, an auxin-like hormone originally considered a synthetic hormone related to plant rooting and used in multiple studies, has now been identified as an endogenous constituent in a wide variety of plants and tissues, although there is not as much information demonstrating its importance as an auxin [34]. It is now known that IBA is an auxin precursor that is converted to IAA in a peroxisomal β-oxidation process. Alternatively, it is suggested that IBA is an auxin storage form [35]. Being a hormone related rather to plant rooting, it is likely that this explains its higher concentration in NI-PSB, waiting to be converted to IAA in a sufficient balance to establish its role in the flowering process of *L. anceps*.

Thus, an inverse IAA/IBA balance in the I-PSB and NI-PSB BGS and higher concentrations of IAA in NI-Leaf and I-PSB CGS, as well as higher IBA in NI-PSB BGS could be probably involved in the inhibition of flowering. Because of the influence of cold treatment on flowering, Zhang et al. [36] discovered that high levels of IAA and ZR controlled the vegetative growth phase and floral induction phase in *Phalaenopsis*. In our research, it seems that a low concentration of IAA in I-Leaf, but high in I-PSB, could apparently play an important role in flowering of *L. anceps*. In addition, de Melo Ferreira [37] proposed that TDZ action may include both auxins and CKs in the floral transition of *Dendrobium* Second Love in vitro. The impact of auxin flow to the floral bud initiation site, nevertheless, is unclear in the literature [38], although some studies show their involvement in flowering. For example, Zhang et al. [33] discovered that IAA increased by 581% in stem apices from the vegetative to the bud stage of inflorescence buds of *Agapanthus praecox* ssp. *orientalis*, with the most significant changes occurring during flowering. These authors propose that IAA is involved in the differentiation and development of each floral organ, and that IAA signaling is involved in pedicel and corolla elongation, as well as slightly delaying flowering. In support of this idea, the effect of exogenous GA_3_ on endogenous hormone levels in peony (*Paeonia suffruticosa*) plants was investigated, and the authors found that it stimulated the synthesis of endogenous GA_3_ and IAA while decreasing abscisic acid (ABA) levels. Additionally, they found that GA_3_ significantly increased flower bud development, vegetative growth, and improved flowering quality [17].

Based on the above ideas, it is thought that the pseudobulbs could act as a reservoir organ for water, nutrients, and carbohydrates [39,40,41] but also, as Zotz [40] mentions, the importance of the backshoots in providing water, carbohydrates, and nutrients to new growth structures in the next cycle in *Dimerandra emarginata* (Orchidaceae); the data from our study suggest that the pseudobulb plays an important role in regulating flowering in *L. anceps* as a reservoir through the accumulation of endogenous hormones, which is supported in part by Chen et al. [42], who establish a correlation between transcriptome and metabolome in pseudobulbs of *Bletilla striata* (Orchidaceae), shedding light on the synthesis pathways of bioactive substances, including hormones.

### 3.2. Total Sugars

Doses of 400 mg L^−1^ GA_3_ in interaction with CGS significantly increased the concentration of sugars. Likewise, the single effects of some exogenous GA_3_ doses tended to increase sugar concentration relative to no GA_3_ in leaves and pseudobulbs of flowering and non-flowering induced plants, but only doses 800 in NI-Leaf and 400 mg L^−1^ GA_3_ in NI-PSB increased sugars statistically (Figure 7B). Giberrellins influence source-sink metabolism and play an important role in carbohydrate partitioning [43]. In the data presented here, it was also observed that the concentration of sugars in I-Leaf in both BGS and CGS was lower than in NI-Leaf, implying that the former is destined for flowering. In this regard, Yong and Hew [44] demonstrated that the current shoot of the thin-leaved sympodial orchid hybrid *Oncidium goldiana* are the main source of photoassimilates for the current shoot inflorescence, while the leaves of the connected back shoots are sources of secondary photoassimilates. While there was no significant difference in sugars between BGS and CGS in I-PSB, a relative inverse pattern of sugars was observed between I-PSB and NI-PSB, implying that the CGS of I-PSB, with lower sugar concentrations, is probably also allocating them to flowering, and this energy consumption is supported by a higher concentration of sugars in the BGS during induction.

Indirect support for this idea is that which indicates lower pseudobulb dry matter in the defoliated current shoot indicates some degree of remobilization of storage reserves for inflorescence production [44]. In NI-PSB, the opposite effect was observed, with CGS having considerably greater sugar concentrations than BGS. The accumulation of sugars by photosynthesis is vital for orchid flowering [45]. The authors add that flowering of *Oncidium* is rather by the autonomous route and is closely linked to the nutritional status of the pseudobulb. Similar to our findings, Kozłowska et al. [46] discovered that in *Zantedeschia* ‘Black Magic’, the carbohydrate level increased in response to GA_3_ treatments independently of earlier stimulation of stem emergence. The authors observed that during flowering, there is a GA-effect on the transport of assimilates to the sink organs, implying that stem growth stimulates photosynthetic activity of GAs, which promotes flowering performance of callas. Furthermore, Mornya et al. [47] discovered higher levels of IAA and GA_3_, sucrose, and reducing sugars in buds of flowering tree peony cv. Ao-Shuang, which could influence the induction of flowering in autumn. The authors hypothesize that peony flowering could be influenced by a variety of hormonal signals and sugars.

Because of different doses of GA_3_, different balances in the endogenous hormone and sugar concentrations in the structures analyzed were observed, as well as a differential distribution in leaves and pseudobulbs during their induction or non-induction to flowering. Hormone interactions rather than individual hormones control growth and development, and the relative concentration may be more important than the concentration of any specific hormone [32].

## 4. Materials and Methods

### 4.1. Plant Material

Adult plants in reproductive stage from in vitro propagation (Orquídeas Río Verde, Temascaltepec, Mexico) of *Laelia anceps* subsp. *anceps* (hereafter abbreviated as *Laelia anceps*), a sympodial thick-leaved epiphytic orchid with 6 ± 1 linked growth structures (consisting of leaves, pseudobulbs, roots, and rhizomes), were transplanted into 1 L translucent pots in March 2018. A substrate mix was used with 60% pine bark (Vigoro^®^; medium grade), 20% zeolite, and 20% peatmoss (CP:Z:PM; *v*:*v*:*v*). They were kept in the adaptation phase in this container and arranged nine months later for distribution in flowering induction treatments. Four months before their distribution to treatments (from January to April 2019), they were maintained with weekly fertilization Foresta^®^ S.A. de C.V., 2 g L^−1^, (NPK 20:20:20), for vegetative shoot growth.

### 4.2. Inductive Treatments and Experimental Design

Plants with at least one vegetative growing shoot of 18 cm of average length with immature pseudobulb and leaf, closed leaf blade, and no visible inflorescence were chosen. Fertilization with Foresta^®^ was changed to Phytophos-K (Phyto-nutrimentos^®^, S.A. de C.V.), 1 g L^−1^ (NPK 10:21:36) with weekly applications. Pack Hard (Innovak Global^®^) 2 mL L^−1^ (Ca 8%, B 0.25%, polyhydroxylic acids 6%, *w*/*w*) were also applied weekly, beginning in 15 May 2019.

The following doses of gibberellic acid (GA_3_; Gibiotin^®^) were tested: 0, 400, 600, 800, and 1000 mg L^−1^. Three weekly applications of 30 ml foliar sprays to the entire plant were made at ambient temperatures ranging from 23 to 25 °C. Distilled water was used in the preparation of nutritive solutions, with growth regulators, and for plant watering.

**Figure 9:**
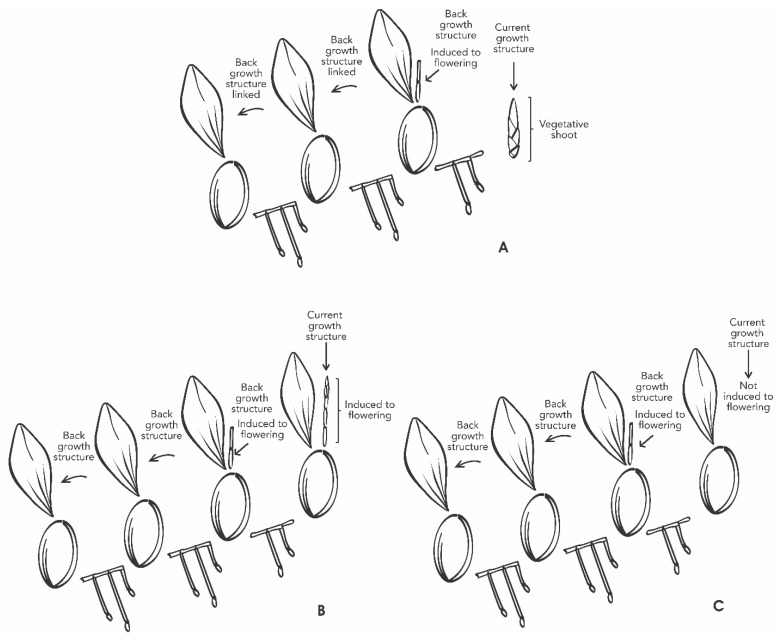
Schematic overview of *Laelia anceps* subsp. *anceps* plants showing back and current growth structure linked, with vegetative shoot (**A**), induced to flowering (**B**), and non-induced to flowering (**C**). The CGS may or may not be induced, despite the reproductive stage of the BGS.

A factorial experiment was designed with the following study factors: (i) GA_3_ (with five levels previously described, (ii) back and current growth structures linked in the same plant (BGS and CGS) (Figure 9). Each plant pot was used as the experimental unit, and each treatment had eight repetitions.

### 4.3. Growth Conditions

For vegetative shoot stimulation, plants were maintained under greenhouse conditions with the following environmental data (HOBO Data Logger Onset and HOBOware PRO^®^; Onset^®^; Bourne, MA, USA): maximum and minimum temperature, 36 and 15 °C; average temperature, 26 °C; maximum and minimum photosynthetic photon flux density in the daytime (PPFD), 189 and 81 µmol m^−2^ s^−1^; maximum and minimum relative humidity (RH), 90 and 40%, respectively.

For the flowering inductive treatments, orchids were placed in a plant growth controlled-environment chamber with LED light when vegetative buds had the indicated size (18 cm average length), and whose leaf and pseudobulb differentiation was barely visible (to avoid floral induction before treatments); the material was entered into a controlled-environment chamber with the following conditions: red and blue LED lamps, ratio 90:10%), with a photosynthetic photon flux (FFF) of 120 µmol m^−2^ s^−1^, with photoperiod 12/12 h, and temperature 28/12 ± 1 °C. GA_3_ doses were applied to the plants two days after they were placed in the chambers.

### 4.4. Data Collection

Three flowering induced and three non-induced plants were chosen for each treatment. Plants with induced structures were sampled when the visible inflorescence had reached a size of 5 cm. The BGS and CGS were removed from each plant, and the latter were classified as induced or non-induced to flowering. Each structure was divided into two parts: leaves and pseudobulbs. Each plant organ was fragmented, and half of its fresh weight was used to calculate total sugars and the other half to analyze endogenous hormones. The material was kept at −70 °C in a deep freezer (ThermoScientific, Revco Elite Series; Waltham, MA, USA) while the corresponding analyses were performed.

### 4.5. Determination of Endogenous Plant Hormones

A previously frozen sample of the material was placed in a lyophilizer (Labconco) for 72 h. For the extraction of hormones, the method proposed by [48] was followed. Endogenous hormone analysis and quantification was done by diode array detector according to Ricker (2000) [Available online: Plant Hormones Rapid Gradient Elution Separation (Agilent Plant Hormones rapid gradient elution separation: Free Download, Borrow, and Streaming : Internet Archive, accessed on 20 January 2022)]. Thus, 100 mg of each sample were weighed into 2.0 mL conical-bottom plastic tubes in triplicate, followed by 500 µL of extraction solution. After shaking the tubes at 100 rpm for 30 min at 4 °C, 1 mL of HPLC grade methylene chloride was added and stirred for another 30 min. For 5 min, the tubes were centrifuged at 13,000 rpm. 900 µL of the organic phase was transferred to an amber vial and dried under a stream of nitrogen gas until the volume reached 100 µL. Following that, 500 µL of HPLC grade methanol was added. A volume of 100 µL was injected into the liquid chromatograph. High performance liquid chromatography (HPLC) was used to determine endogenous hormones listed below. The liquid chromatograph (Agilent Technologies model 1100) was equipped with an automatic injector mod. 1200 and a Model 1100 Diode Array Detector, Agilent Technologies (Santa Clara, CA, USA) Rx/SB-C8 Rapid Res 4.6 × 75 column, with the mobile phase consisting of the following solvents: A (80%): 0.1% trifluoroacetic acid and B (20%): 0.1% trifluoroacetic acid in acetonitrile. The flow was 2 mL min^−1^ the temperature was 60 °C, the injection volume was 100 µL, and the detector was set at 254 nm.

The hormone profile involved the identification of some major hormones, in their free forms, related to flowering. Thus, among the cytokinins (CKs), the following were analyzed: zeatin (ZEA), a mixture of *cys* and *trans* isomers, as well as trans-zeatin (T-ZEA), a highly active form in plant tissues. In addition, kinetin (KIN), a CK little studied to which important properties are currently attributed, and whose endogenous role in flowering has been scarcely reviewed. Among the auxins, indoleacetic acid (IAA) and indole-3-butyric acid (IBA) were determined, the latter also with poorly understood functions in plants and not studied in flowering. In addition, endogenous GA_3_ and abscisic acid (ABA) were also assessed. Each endogenous hormone was identified and quantified using a standard curve, in accordance with the growth regulator standards (Sigma-Aldrich; Saint Louis, MO, USA).

### 4.6. Determination of Total Sugars

The concentration of total soluble sugars was determined using the method described by Southgate [49] with anthrone, sulphuric acid, and 80% ethanol. The absorbance was measured at 620 nm using a spectrophotometer (Jenway, 6715 UV/Vis; Cole-Parmer, Vernon Hills, IL, USA). For the calibration curve, glucose was used as standard.

### 4.7. Statistical Analysis

The analyses were conducted separately for plants induced and non-induced to flowering, considering the different doses of GA_3_ applied exogenously and the two growth structures, with the three replications previously indicated. A 2-way ANOVA and the Tukey mean comparison test (*p* ≤ 0.05) were performed on the obtained data (SAS/STAT 9.4, SAS, Institute Inc., Cary, NC, USA).

## 5. Conclusions

Based on our findings, the endogenous hormone profile analyzed in the samples of *Laelia anceps* allows us to conclude some relevant aspects: (i) GA_3_ doses sprayed to current growth structures significantly increase the concentration of kinetin; (ii) exogenous GA_3_ increased endogenous GA_3_ in induced and non-induced flowering leaves and pseudobulbs; (iii) higher concentration of endogenous hormones, GA_3_ and both cytokinins and auxins, in pseudobulbs compared to leaves; (iv) opposite balances of GA_3_, cytokinins and auxins in both leaves and pseudobulbs induced and not induced to flowering; (v) opposite balances in back and current growth structures.

The sugars concentration allows the conclusion that: (i) low dose, 400 mg L^−1^ GA_3_, in interaction with the current growth structure of pseudobulb non-induced to flowering, displays a hormesis-like effect; (ii) pseudobulb induced to flowering has lower sugars concentration in the current growth structure and higher in the previous growth structure, compared to its similar in the non-induced pseudobulb.

GA_3_, KIN, ZEA, and IAA are thought to play a key role in flowering from the pseudobulb, mainly in the current growth structure, while higher concentration of T-ZEA in the non-induced leaf and IBA in the non-induced pseudobulb are thought to participate in the inhibition of flowering from back growth structure. It is proposed that sugars are remobilized during flowering. This is a first study to look at the endogenous hormone profile with differential distribution in leaves and pseudobulbs, induced and non-induced to flowering, and it is likely that the pseudobulb is a potential reservoir of bioactive substances, such as hormones, that may regulate flowering.

## Figures and Tables

**Table 1:** *p*-value of the study factors for endogenous hormones in leaf and pseudobulb of *Laelia anceps* subsp. *anceps* plants, induced and non-induced to flowering.

Factors	Induced Leaf	Induced Pseudobulb			
	ZEA	ZEA	KIN	IBA	GA_3_
GA_3_	0.7217	0.3456	0.0164 *	0.3643	0.0456 *
GS	0.0260 *	0.0443 *	<0.0001 **	0.0145 *	0.0097 **
GA_3_xGS	0.5921	0.6476	0.0171 *	0.3643	0.3012
**Factors**	**Non-Induced Leaf**	**Non-Induced Pseudobulb**			
	**ZEA**	**KIN**	**IAA**	**IBA**	**GA_3_**
GA_3_	0.4072	0.6835	0.6969	0.5244	0.0209 *
GS	0.0049 **	0.0059 **	0.0157 *	0.0238 *	0.0017 **
GA_3_xGS	0.5475	0.5809	0.9663	0.5210	0.0593

GS: Growth structure; GA_3_: Doses of exogenous gibberellic acid; ZEA: zeatin; KIN: kinetin; GA_3_: gibberellic acid; IAA: indoleacetic acid; IBA: indole-3-butyric acid; * *p* ≤ 0.05 is statistically significant; ** *p* ≤ 0.01 is statistically significant.

**Table 2:** Concentration (ng g^−1^ dry weight) of endogenous gibberellic acid (GA_3_) and kinetin (KIN) in leaf and pseudobulb induced and non-induced to flowering in *Laelia anceps* subsp. *anceps* plants by exogenous GA_3_ application.

	Induced Leaf	Non-Induced Leaf	Induced Pseudobulb		Non-Induced Pseudobulb
GA_3_ DOSES (mg L^−1^)	GA	GA	KIN	GA	GA
0	0.0 ^b^	0 ^b^	7.0 ^b^	5.5 ^b^	6.0 ^b^
400	0.0 ^b^	1.8 ^b^	46.3 ^ab^	71.8 ^ab^	2.7 ^b^
600	7.3 ^b^	47.1 ^a^	46.4 ^ab^	35.5 ^ab^	39.1 ^ab^
800	17.6 ^ab^	37.0 ^ab^	54.6 ^a^	36.2 ^ab^	68.7 ^ab^
1000	41.9 ^a^	24.6 ^ab^	55.9 ^a^	143.0 ^a^	89.3 ^a^

Different letters in each column indicate statistical differences (Tukey, *p* ≤ 0.05; *n* = 3).

**Table 3:** *p*-value of the study factors for total sugars in leaf and pseudobulb of *Laelia anceps* subsp. *anceps* plants, induced and non-induced to flowering.

Factors	Induced		Non-Induced	
	Leaf	Pseudobulb	Leaf	Pseudobulb
GA_3_	0.1120	0.0734	0.0301 *	0.0021 **
GS	0.0037 **	0.0726	0.1250	0.0093 **
GA_3_xGS	0.7213	0.9021	0.8213	0.0001 **

GA_3_: gibberellic acid doses; GS: growth structure; * *p* ≤ 0.05 is statistically significant; ** *p* ≤ 0.01 is statistically significant.

## Data Availability

Not applicable.
